# Effective service coverage of long-term care among older persons in South Korea

**DOI:** 10.1093/ageing/afad120

**Published:** 2023-10-30

**Authors:** Ja-Ho Leigh, Hyejin Lee, Jaehong Yoon, Eun-Jeong Han, Eunok Park, Tong Ryoung Jung, Jotheeswaran Amuthavalli Thiyagarajan, Zee-A Han

**Affiliations:** Department of Rehabilitation Medicine, Seoul National University College of Medicine, Seoul National University Hospital, Seoul, South Korea; National Traffic Injury Rehabilitation Research Institute, National Traffic Injury Rehabilitation Hospital, Yangpyeong-gun, South Korea; Department of Family Medicine, Seoul National University Bundang Hospital, Seongnam, South Korea; Department of Family Medicine, Seoul National University, Seoul, South Korea; Department of Rehabilitation Medicine, Seoul National University College of Medicine, Seoul National University Hospital, Seoul, South Korea; National Traffic Injury Rehabilitation Research Institute, National Traffic Injury Rehabilitation Hospital, Yangpyeong-gun, South Korea; Health Insurance Research Institute, National Health Insurance Service, Wonju, South Korea; College of Nursing, Health and Nursing Research Institute, Jeju National University, Jeju, South Korea; Division of Public Health Emergency Management, Korea Disease Control and Prevention Agency, Cheongju, South Korea; Department of Maternal, Newborn, Child and Adolescent Health and Ageing, World Health Organization, Geneva, Switzerland; Department of Rehabilitation Medicine, Uijeongbu Eulji Medical Center, Eulji University, Uijeongbu, South Korea; Department of Rehabilitation Medicine, College of Medicine, Eulji University, Daejeon, South Korea

**Keywords:** long-term care, activities of daily living, global trends, medical aid, older people

## Abstract

**Background:**

Global population aging, and the accelerated increase in the number of oldest-old adults, over 80 years, has implied a heightened need for long-term care (LTC). We aimed to provide a theoretical care cascade of LTC services to assess publicly funded LTC (Analysis 1) and to investigate the association between the use of LTC insurance (LTCI) and unmet care needs among older people (Analysis 2) in South Korea.

**Methods:**

Analysis 1 used data from the eighth wave (2020) of the Korean Longitudinal Study of Aging (KLoSA), the 2020 National Health Insurance Service LTCI Statistical YearBook and the 2020 National Awareness Survey of LTCI. The care cascade consisted of the target population, service contacts, coverage and outcomes. Analysis 2 used the fifth to eighth waves of KLoSA, and LTCI analysis was based on three groups: not aware, aware but do not use and aware and use. Unmet care needs were defined as the absence of help among older people with care needs.

**Results:**

Among 8,489,208 people aged 65 or older in 2020, 1,368,148 (16.1%) were estimated to want care. Of these, 62.7% (*N* = 857,984) had LTCI service contact and 807,067 (94.1%) of those had used LTCI services in the past year (Analysis 1). Older people who were aware and used LTCI were less likely to report unmet activities of daily living (ADL) (prevalence ratio (PR): 0.34, 95% confidence interval (CI): 0.18–0.66) or unmet instrumental ADL (IADL) needs (PR: 0.27, 95% CI: 0.17–0.43) than those who were not aware (Analysis 2).

**Conclusions:**

This article provides a theoretical cascade to assess LTC provision in South Korea and a preliminary model for other countries. Korea’s LTCI is associated with reduced unmet ADL and IADL needs.

## Key Points

- This article provides a theoretical care cascade to assess long-term care (LTC) provision in South Korea and a preliminary model to hypothetically assess and monitor publicly funded LTC in other countries.
- The care cascade showed a large gap between those wanting care and eligibility for LTC insurance (LTCI), and thus future studies are needed to examine the reasons for this gap.
- Korea’s LTCI is associated with reduced unmet activities of daily living (ADL) and instrumental ADL care needs as well as health gains despite its limitations.

## Introduction

Global population aging and the accelerated increase in the number of oldest-old adults, over 80 years, have implied a heightened need for long-term care (LTC) [1]. According to the World Health Organization (WHO), over two-thirds of older adults need LTC, including support to perform activities of daily living (ADL). With the commencement of the United Nations (UN) Decade of Healthy Ageing (2021–2030), the WHO developed an LTC framework to help countries achieve an integrated continuum, and equitable provision, of LTC [2]. The framework emphasises the need for an integrated continuum of LTC services provided and received in a non-fragmented way, aligned with the needs and values of older people and upholding the principles of integrated care for older people (ICOPE) [3, 4].

In line with global trends, South Korea (Korea) also has a rapidly aging population. The proportion of those aged 65 and over in Korea was 15.7% in 2020 and is expected to exceed 20% by 2025 and 40% by 2050 [5]. The numbers of those 80 years and over are expected to increase particularly steeply, from 3.6% of the total population in 2020 to 4.9% in 2025 and 16.5% in 2050 [5]. Furthermore, urbanisation in Korea has mitigated filial duty embedded in Korean culture, with rates of family support for older parents decreasing from 18.8% in the 1970s to 4.7% in 2020 [6].

These demographic trends have emphasised the importance of a robust LTC system and led to the establishment of Korea’s LTC insurance (LTCI) in 2008. LTCI is legally mandated through the Act on Long-Term Care Insurance for Senior Citizens, with overall responsibility resting with the Ministry of Health and Welfare [7]. Coordination, implementation and monitoring of LTCI are managed by the National Health Insurance Services (NHIS) as the insurer. The NHIS is legally mandated to continuously monitor, assess and improve the quality of the insurance program and LTCI services.

During initial implementation, LTCI focused on providing care for older adults with severe difficulties in independently performing ADL. However, it has slowly expanded to include those with less severe care needs. It has made efforts to be more inclusive of the community, provide continuity of care and be more attuned to individual needs.

In this paper, we estimate the cascade of care needs, service provision status and health outcomes of older people in Korea to explore effective coverage of long-term care provision (Analysis 1). Furthermore, we analysed a nationally representative Korean Longitudinal Study of Aging (KLoSA) to examine the care needs of older people, with a focus on ADL/Instrumental ADL (IADL), and whether the current use of LTCI is associated with reducing the unmet care needs of older people (Analysis 2). This is the first article to report on a theoretical cascade to assess long-term care provision in Korea and provides a preliminary model to hypothetically assess and monitor publicly funded LTC in other countries.

## Methods

### A theoretical cascade estimating care needs, service provision and health gains of older people in Korea through publicly funded LTC (Analysis 1)

#### Study population

We used three datasets to develop a cascade of health gain through LTCI for older people wanting care: the eighth wave of the Korean Longitudinal Study of Aging (KLoSA), the 2020 NHIS LTCI Statistical YearBook and the 2020 National Awareness Survey of LTCI. KLoSA was designed to explore the social, economic, psychological and health status of older adults to formulate socioeconomic policies. The survey was conducted biennially with a representative sample of community-dwelling older people (45 years or older) in Korea by the Korea Labor Institute. A total of 10,254 respondents were recruited during the first wave of KLoSA, and all KLoSA data were publicly available online (survey.keis.or.kr). First, we utilised data from the eighth wave (2020) to match the timing of the other datasets (2020), including people 65 years and older in Analysis 1 (*N* = 4,405). Second, we used the 2020 LTCI Statistical Yearbook, which was based on LTCI claims from NHIS. The NHIS database includes almost all individuals residing in Korea [8] and the YearBook has been published annually to support welfare and health care policy for older people. Finally, we utilised the LTCI National Awareness Survey, designed to investigate public awareness of LTCI for system improvement. The nationwide survey was conducted with the general population (*N* = 1,500), caretakers (*N* = 1,000) and health providers (*N* = 1,000) in 2020, and the survey for caretakers was included in Analysis 1.

#### Measurement

To identify older people wanting care, ADL and IADL were assessed in KLoSA [9, 10]. ADL was composed of seven items (dressing and undressing, personal hygiene, bathing, eating, transferring from bed, toileting and voluntarily controlling urinary and faecal discharge). IADL consisted of 10 items (decorating oneself, cleaning, preparing meals, doing laundry, mobility, public transportation, shopping for groceries, managing money, using the telephone and taking medications). Respondents answered ‘no help needed’, ‘partial help needed’ and ‘help needed.’ Respondents indicating ‘no help needed’ to all domains of ADL or IADL were defined as those with no ADL or IADL care needs. Those indicating ‘partial help needed’ and ‘help needed’ were defined as those with ADL or IADL care needs [11].

To measure health gains, a questionnaire for caretakers was used: how has the overall health status of older people changed after using LTCI? The respondents could answer ‘best’, ‘better’, ‘similar’, ‘worse’ and ‘worst.’ The ‘best’ and ‘better’ responses were defined as health gains due to LTCI services.

#### Statistical analysis

For the cascade of care needs, we defined the target population, service contact, coverage and outcome. First, we considered older people wanting care as the target population, estimating the number through two steps. We calculated the number of older people with ADL or IADL care needs by multiplying the number of older people and the proportion of those with ADL or IADL care needs. Older people were defined as 65 years and older, and the number of older people was based on the NHIS YearBook. The proportion of those with ADL or IADL care needs was estimated using KLoSA. KLoSA provides both cross-sectional and longitudinal weights for individuals in every wave. We applied the 2020 cross-sectional weights to estimate a nationally representative proportion of those with care needs. To consider the probability of exclusion of older people in institutions with ADL/IADL care needs, we added the number of residents in LTC institutions (both LTC facilities and LTC hospitals) to the number of older people with ADL or IADL care needs. The number of residents in LTC institutions was taken from the 2020 NHIS YearBook (*N* = 146,998).

Second, those who were assessed as in need of LTCI and those who received services through LTCI were defined as service contacts and coverage, respectively. The number of service contacts and coverage was derived from the NHIS YearBook, based on the NHIS claim database. Finally, by multiplying coverage and the proportion of health gain, we estimated the outcome.

### Estimating the association between LTCI use and unmet care needs (Analysis 2)

#### Study population

We utilised data from the fifth to eighth waves because questions on LTCI have been included since the fifth wave. A total of 2,769 observations from 1,581 adults were analysed. The inclusion criteria were (i) 65 years and older; (ii) needed help in ADL or IADL; and (iii) did not have missing information on ADL or IADL, health insurance status and other covariates. The requirement for informed consent was waived because the study was based on open datasets available to the public.

#### Measurement

LTCI use was assessed using three questions: (a) ‘Are you aware of LTC insurance?’ (b) ‘Have you ever applied for LTC insurance?’ and (c) ‘Do you currently use LTC insurance?’ Respondents could answer ‘yes’ or ‘no.’ Based on the responses to the questions, respondents were classified into three groups: (1) ‘not aware’ meant they were unaware of LTCI, (2) ‘aware but do not use’ indicated that they were aware of LTCI but did not use the insurance and related services and (3) ‘aware and use’ indicated that they were aware of and used LTCI.

Unmet care needs were defined through two steps. First, to assess care needs, we utilised ADL and IADL and defined care needs in the same way as in Analysis 1. Second, people with care needs were asked whether they had caretakers. Respondents who answered ‘absence of help’” were defined as those with unmet ADL and IADL care needs, while the others were considered to have their care needs met.

We considered demographic variables (age, sex, marital status, region, living alone and year), socioeconomic status (educational level and employment status) and health-related variables (self-rated health; cancer, chronic lung disease, cardiac disease and cerebrovascular disease diagnoses; and insurance status) as covariates for each wave (5th–8th). The analysis of the covariates is explained in Appendix 1.

#### Statistical analysis

For descriptive analysis, we performed the Rao–Scott design-adjusted chi-square test with sample weights to compare covariates across unmet care needs. We used the cross-sectional weights from each wave to estimate the proportion of those with unmet needs in the weighted population. Association analyses were performed to investigate the influence of LCTI on unmet care needs. To consider the autocorrelation of individuals, we applied the random intercept modified Poisson model with robust standard error after adjusting for covariates, including disease diagnosis. Furthermore, we examined the age-specific association between health insurance information and unmet care needs because age groups might represent different life stages: early older age (65–74 years old), middle older age (75–84 years old) and late older age (≥85 years old). All analyses were performed using the STATA/MP version 17.0 (Stata Corp., College Station, TX, USA).

## Results

### A theoretical cascade estimating care needs, service provision and health gains of older people in Korea through publicly funded LTC (Analysis 1)

**Figure 1:**
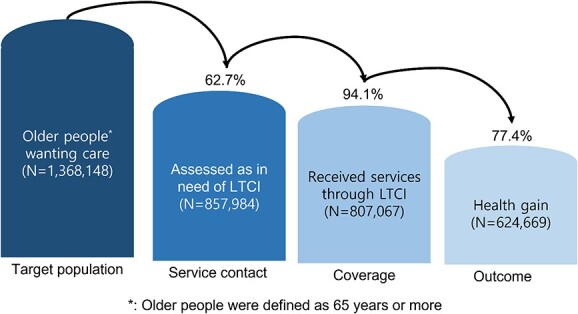
Cascade of health gain through LTCI for older people wanting care.

As of 2020, there were 8,489,208 people aged 65 or older in South Korea, and older people wanting care were estimated at 1,368,148 (16.1%). Of these, 62.7% (*N* = 857,984) had LTCI service contact and 807,067 (94.1%) of those had used LTCI services in the past year. Among those who used the LTCI service, 624,669 (77.4%) responded that their subjective health status had improved compared to that before using the service (Figure 1).

### Estimating the association between LTCI use and unmet care needs (Analysis 2)

The weighted population of older people was 3,989,521, and the weighted proportion of unmet care needs for ADL or IADL was 29.7% (95% CI: 27.8–31.7). Unmet care needs were prevalent among people who were early old age (36.4%, 95% CI: 32.3–40.8), male (32.6%, 95% CI: 29.5–35.9), employed (47.7%, 95% CI: 40.7–54.8) and who were aware but did not use LTCI (33.6%, 95% CI: 30.6–56.8) (Table 1). Furthermore, older people who had subjectively good health status (42.8%, 95% CI: 38.8–46.9) and were not diagnosed with cerebrovascular disease (31.5%, 95% CI: 29.4–33.7) reported that they needed more care.

**Table 1:** Weighted percentage of study population and unmet care needs by covariates from 2014 to 2020

	Total population^a^ (*N* = 3,989,521)	Unmet care needs^b^	*P*-value^c^
	Weighted percentage of total	% (95% CI)	
Total	100	29.7 (27.8, 31.7)	
Age			<0.001
65–74 years	31.9	36.4 (32.3, 40.8)	
75–84 years	42.2	30.3 (27.6, 33.2)	
85 years ≤	25.9	20.3 (17.7, 23.2)	
Sex			0.011
Male	43.7	32.6 (29.5, 35.9)	
Female	56.3	27.4 (25.0, 29.9)	
Marital status			0.590
Married	55.2	30.2 (27.6, 32.9)	
Others	44.8	29.1 (26.2, 32.1)	
Region			0.519
Metropolis	41.1	31.0 (27.9, 34.2)	
Small or medium city	30.5	28.4 (24.9, 32.1)	
Town or village	28.3	29.2 (25.9, 32.8)	
Living alone			0.001
No	78.7	27.9 (25.8, 30.1)	
Yes	21.3	36.2 (31.6, 40.9)	
Year			0.005
2014	25.0	24.9 (21.6, 28.6)	
2016	26.0	32.3 (28.6, 36.1)	
2018	22.3	27.2 (23.5, 31.3)	
2020	26.8	33.6 (29.4, 38.0)	
Education level			<0.001
Primary school or less	65.1	26.0 (23.8, 28.2)	
Middle school	13.2	35.9 (30.0, 42.2)	
High school	15.2	39.8 (34.4, 45.5)	
College or more	6.47	30.4 (22.1, 40.3)	
Employment status			<0.001
Employed	10.4	47.7 (40.7, 54.8)	
Retired & others	89.6	27.6 (25.6, 29.6)	
Self-rated health status			<0.001
Bad	72.2	24.6 (22.5, 26.8)	
Good	27.8	42.8 (38.8, 46.9)	
Cancer diagnosis			0.603
No	93.7	29.8 (27.8, 31.9)	
Yes	6.27	27.8 (21.2, 35.5)	
Chronic lung disease diagnosis			0.106
No	94.7	29.9 (27.9, 32.0)	
Yes	5.32	25.0 (18.3, 33.3)	
Cardiac disease diagnosis			0.341
No	84.0	30.1 (28.0, 32.3)	
Yes	16.0	27.4 (22.8, 32.6)	
Cerebrovascular disease diagnosis			<0.001
No	83.2	31.5 (29.4, 33.7)	
Yes	16.8	20.6 (16.5, 25.4)	
Insurance status			0.697
National Health Insurance beneficiary	88.3	29.5 (27.5, 31.6)	
Medical aid recipient	11.7	30.8 (25.1, 37.1)	
LTC insurance			<0.001
Not aware	48.5	29.9 (27.2, 32.8)	
Aware but do not use	43.3	33.6 (30.6, 36.8)	
Aware and use	8.21	7.47 (4.2, 12.9)	

^a^Older people who needed help with ADL or IADL.

^b^Unmet care needs for ADL or IADL.

^c^ *P*-value of the Rao–Scott design-adjusted chi-square test comparing the age groups across the different groups.

Older people who were aware of LTCI were less likely to report unmet ADL needs, whether they used (prevalence ratio (PR): 0.34, 95% CI: 0.18–0.66) or did not use the services (PR: 0.66, 95% CI: 0.50–0.88), than those who were not aware (Table 2). For IADL needs, being aware of LTCI and using the services was significantly associated with fulfilment of care needs (PR: 0.27, 95% CI: 0.17–0.43).

For age-specified relationships, Appendix 2 shows that unmet ADL care needs were significantly related to awareness and use of LTCI among middle old age respondents (PR: 0.09, 95% CI: 0.01–0.67) and to awareness without use of LTCI among late old age respondents (PR: 0.46, 95% CI: 0.25–0.85). Although older people who were aware of LTCI but did not use the service had lower unmet ADL needs than those who were unaware, the association was not statistically significant. Additionally, early old age (PR: 0.11, 95% CI: 0.02–0.81), middle old age (PR: 0.12, 95% CI: 0.04–0.38) and late old age (PR: 0.45, 95% CI: 0.26–0.78) respondents who were aware of LTCI and using the services were less likely to report unmet IADL needs than those who were unaware of the service.

## Discussion

There have been previous studies analysing and measuring care cascades and effective coverage in various health specific areas including maternal, child and adolescent health [12, 13]. However, this study is the first to explore effective service coverage for LTC. As in previous studies, measuring contact with services is not sufficient to indicate improvement of health outcomes among users. Measuring beyond utilisation of LTC services using coverage cascades can assess not only performance of LTCI but also the sequences of interactions between population in need and the LTC system, including areas where bottlenecks in service provision exist. As South Korea aspires to improve universal health coverage for older persons, the care cascade presented in this paper may allow national governments to set targets to improve coverage of LTC and achieve meaningful health outcomes through LTCI.

According to our analysis, 16.1% of older people were wanting care services. Among those wanting care, 62.7% were eligible for LTCI, of which 94.1% used LTCI services and 77.4% reported health gains due to use of LTCI services (Figure 1).

The reasons for this existing large gap between those wanting care and eligibility of LTCI is an area that should be studied more closely. One possible explanation for this phenomenon is that, first, the Korean LTCI system requires individuals to apply for LTCI service contracts in order to receive benefits, and older adults who are not well informed about LTCI may not have applied for it [14]. Second, it is known that the distribution of LTC hospitals, nursing homes and home care agencies in a given region can affect LTCI service contact, as regions with fewer service providers may have lower LTCI service contact rates [15].

Our analysis also revealed about one-fourth not reporting health gains after using LTCI services. This may be related to the quality of the services provided through LTCI and may imply the need to improve quality of such services. Currently, the NHIS oversees the quality of LTC services provided through the LTCI [16]. For in-home services and aged-care facility care, indicators were defined to monitor domains that included (i) user satisfaction, (ii) processes involved in providing services, (iii) operation of LTC institutions that provide LTC services, (iv) LTC provider competencies, (v) considerations for upholding the rights and dignity of older people and (vi) environment and safety [17]. However, a criticism has been that quality management is based on efficiency rather than person-centeredness [18]. In response to such criticism, specific indicators have been incorporated to monitor person-centeredness, community involvement and continuity of care. There are 50 indicators monitoring long-term facilities, including those monitoring whether comprehensive, person-centred assessment was performed at the beginning and periodically thereafter, whether efforts have been made to enhance the interaction of residents with family and community and whether resident and caregiver feedback is reflected in service provision and facility improvements. Among the 24 indicators for home-visit care, indicators that monitor whether personalised LTC plans have been reflected when contracting for LTC services, whether such personalised LTC plans have also been shared and agreed with the user, whether user needs are periodically reassessed and whether periodic care/case management meetings are held are examples of enhancing and monitor person-centred integrated care [19].

**Table 2:** Association between LTC insurance and unmet care needs among people with care from 2014 to 2020

LTC insurance		ADL (*N* = 1,120)			IADL (*N* = 2,736)	
	Total *N* (%)	Unmet needs 175 (15.2)	PR (95% CI)	Total *N* (%)	Unmet needs 762 (27.9)	PR (95% CI)
Total						
Not aware	591 (51.2)	111 (18.8)	1 (ref)	1,332 (48.7)	377 (28.3)	1 (ref)
Aware but do not use	403 (34.9)	55 (13.7)	066^a^ (0.50–0.88)	1,185 (43.3)	369 (31.1)	0.99 (0.88–1.12)
Aware and use	161 (13.9)	9 (5.6)	0.34^a^ (0.18–0.66)	219 (8.0)	16 (7.3)	0.27^b^ (0.17–0.43)

LTC, long-term care; ADL, activities of daily living; IADL, instrumental activities of daily living; PR, prevalence ratio; CI, confidence interval; ref, reference.

Results of multiple regression after adjusting for age, sex, marital status, region, living alone, year, educational level, employment status, self-rated health, cancer diagnosis, chronic lung disease diagnosis, cardiac disease diagnosis, cerebrovascular disease diagnosis and insurance status.

^a^<0.01.

^b^0.001.

Our results about the proportion of unmet care needs for ADL or IADL in Korea were inconsistent with those of other countries. In a Chinese study of the oldest people, aged over 80 years, unmet LTC needs ranged from 53.1 to 59.5% [20]. In a study of community-dwelling older individuals over 75 in New Zealand, 34% reported undermet or unmet needs. Specifically, undermet or unmet needs for heavy housework were the most common [21]. In a study of older Indian Americans in the USA, 49% had one or more ADL or IADL problems, and 71.9% needed help. Unmet needs occurred in 34.4% [22]. In a Swiss study, 12% had care needs with one or more IADL problems, similar to ours [23]. The reason for these differences is that the definition of care needs differs from study to study, previous studies were conducted on the oldest older adults [20, 21] and the rates of ADL and IADL problems differed according to race [22].

In previous studies, factors related to unmet care needs were old age, low income, living alone, depression [11], many ADL or IADL disorders and lack of social support [22]. In our study, unmet care needs were higher in those aged 60–69 years, males, high school graduates, those with a job, those with good health status and those who were aware of LTCI but did not use it. Although the reason for these results is unclear, it is thought that people with a relatively high socioeconomic status are not eligible for the subsidy scheme [24] and that the quality of LTCI services is unsatisfactory [25]. Furthermore, the results showed that older people without cerebrovascular disease were more likely to have unmet needs compared to those with cerebrovascular disease. This might be due to differences in the accessibility of LTCI in Korea. Geriatric illnesses, including dementia and cerebrovascular disease, were the most common among beneficiaries of LTCI in Korea [26]. Additionally, when they were aware of LTCI, the proportion of LTCI usage among older people with cerebrovascular disease was higher than that among older people with other diseases in our data. This could imply that older people with cerebrovascular diseases received LTCI more than those with other diseases. Therefore, older people with cerebrovascular disease could be less likely to report unmet care needs in Korea.

### Limitations and strength

This study had some limitations. First, there was an overlap between the KLoSA and the LTCI Statistical Yearbook for estimating older people wanting care (target population). According to the Korea Health Promotion Institute, the proportion of residents in LTC institutions was 2.9% among those 65 years or older in 2014 [27]. However, the proportion of residents in LTC institutions was 0.4% in our data (KLoSA). Therefore, the results of the cascade had to be carefully interpreted. Second, we could not establish a temporal order between current use of LTCI and unmet care needs. Analysis 2 was designed so that the year of current use of LTCI and unmet care needs were the same because a continuously collected population was too small (*N* = 653 people) for Analysis 2. Future studies are needed to explore the causal association using a longitudinal design. Third, there could be selection bias because older people with a vulnerable status for ADL/IADL issues, and unmet care needs, might be excluded from the KLoSA. For example, although the proportion of residents in LTC institutions was 2.9% among older people in Korea, those in KLoSA were 0.4%.

Nevertheless, to the best of our knowledge, this is the first study to estimate the theoretical cascade of care needs, service provision status and health outcomes of older people in Korea. Furthermore, the study examined the relationship between current use of LTCI and unmet care needs in Korea.

## Conclusion

Korea’s LTCI is associated with reduced unmet ADL and IADL care needs, and substantial health gains, despite its limitations. However, Korea has realised that providing fragmented services to people with high ADL/IADL care needs has limitations in preventing functional deterioration or institutionalisation. During the decade since LTCI implementation, Korea has continuously revisited its system to support providing and monitoring of more integrated and person-centred LTC in hopes of improving health outcomes for those receiving LTCI services. Korea has also realised the importance of community resources and the role of local governments in achieving sustainability and equitable LTC provisions. In response to this realisation, Korea has initiated new community-based approaches to complement existing LTCI services. It includes an integrated and comprehensive evaluation system that reflects both social and medical care needs. Despite these efforts, as the most rapidly aging country in the world [28], Korea also needs support from the international community to achieve a truly integrated continuum of LTC, as defined by the WHO Framework on LTC [2].

## Supplementary Material

aa-23-0353-File002_afad120
