# Supplementary material for: Effective service coverage of long-term care among older persons in South Korea
Source: Age Ageing. 2023 Oct 30;52(Suppl 4):iv162–9. doi: 10.1093/ageing/afad120 (PMC10615041; doi:10.1093/ageing/afad120)
Supplement: aa-23-0353-File002_afad120 [file aa-23-0353-file002_afad120.docx]

World Health Organisation: *Measurements of Healthy Ageing*

Effective Long-term Care Service Coverage for Older Persons in South Korea

**SUPPLEMENTARY DATA:**

- **Appendix 1.** Covariates included in regression analysis
- **Appendix 2.** Results of age-specified relationship between LTC insurance and unmet care needs among people with care from 2014 to 2020

**Appendix 1. Covariates included in regression analysis**

Age was divided into three categories (60-69, 70-79, ≥80 years old) and sex was classified into male and female. Marital status was divided into currently married and others, which included never married, widowed, and divorced. Region was coded into three groups (metropolis, small or medium city, and town or village). For living alone, people without household members were considered as “yes,” and the others were classified as "no." The year was considered a dummy variable. Education level was divided into four categories (primary school graduate or less, middle school graduate, high school graduate, and college graduate or more). Employment status was coded into two groups (employed, and retired or other). Self-rated health was assessed with a five-scale response to the question “How would you rate your overall health?” Responses ranged from “very good” (score 1) to “very poor” (score 5) and were dichotomized into good health (for responses 1-3) and poor health (for responses 4-5). Disease diagnoses, including cancer and chronic lung, cardiac, and cerebrovascular disease, were assessed in the first wave and, from the second wave, diagnosis and symptoms since the last survey were measured. We created the disease diagnosis in each wave by combining the responses on disease diagnosis at the first wave with the responses on diagnosis and symptoms since the last survey. Insurance status was classified into two categories (insurance beneficiaries and medical aid recipients).

| Variables | Categories |
| --- | --- |
| Age | 60-69 years old |
|  | 70-79 years old |
|  | ≥80 years old |
| Sex | male |
|  | female |
| Marital status | currently married |
|  | others (never married, widowed, or divorced) |
| Region | metropolis |
|  | small or medium city |
|  | town or village |
| Living alone | yes |
|  | no |
| Year | 2014 |
|  | 2016 |
|  | 2018 |
|  | 2020 |
| Education level | primary school or less |
|  | middle school graduate |
|  | high school graduate |
|  | college graduate or more |
| Employment status | employed |
|  | retired & others |
| Self-rated health status | bad |
|  | good |
| Cancer diagnosis | no |
|  | yes |
| Chronic lung disease diagnosis | no |
|  | yes |
| Cardiac disease diagnosis | no |
|  | yes |
| Cerebrovascular disease diagnosis | no |
|  | yes |
| Insurance status | insurance beneficiary |
|  | medical aid recipient |

**Appendix 2. Results of age-specified relationship between LTC insurance and unmet care needs among people with care from 2014 to 2020**

| Long-term care insurance |  | ADL (N=1,120) | |  |  | IADL (N=2,736) | |
| --- | --- | --- | --- | --- | --- | --- | --- |
|  | Total N (%) | Unmet needs 175 (15.2) | PR (95% CI) |  | Total N (%) | Unmet needs 762 (27.9) | PR (95% CI) |
| Early older age |  |  |  |  |  |  |  |
| Not aware | 91 (45.7) | 20 (22.0) | 1 (ref) |  | 253 (40.7) | 89 (35.2) | 1 (ref) |
| Aware but do not use | 90 (45.2) | 23 (25.6) | 0.82 (0.51-1.32) |  | 342 (55.0) | 126 (36.8) | 0.92 (0.73-1.16) |
| Aware and use | 18 (9.1) | 0 (0.0) | . |  | 27 (4.3) | 1 (3.7) | 0.11* (0.02-0.81) |
| Middle older age |  |  |  |  |  |  |  |
| Not aware | 236 (50.2) | 45 (19.1) | 1 (ref) |  | 600 (49.6) | 181 (30.2) | 1 (ref) |
| Aware but do not use | 170 (36.2) | 21 (12.4) | 0.66 (0.41-1.08) |  | 522 (43.1) | 171 (32.8) | 1.05 (0.88-1.24) |
| Aware and use | 64 (13.6) | 1 (1.6) | 0.09* (0.01-0.67) |  | 88 (7.3) | 3 (3.4) | 0.12*** (0.04-0.38) |
| Late older age |  |  |  |  |  |  |  |
| Not aware | 264 (54.3) | 46 (17.4) | 1 (ref) |  | 479 (53.0) | 107 (22.3) | 1 (ref) |
| Aware but do not use | 143 (29.4) | 11 (7.7) | 0.46* (0.25-0.85) |  | 321 (35.5) | 72 (22.4) | 0.93 (0.70-1.23) |
| Aware and use | 79 (16.3) | 8 (10.1) | 0.59 (0.29-1.20) |  | 104 (11.5) | 12 (11.5) | 0.45** (0.26-0.78) |

LTC, long-term care; ADL, activities of daily living; IADL, instrumental activities of daily living; PR, prevalence ratio; CI, confidence interval; ref, reference.

Results of multiple regression after adjusting for sex; marital status; region; living alone; year; education level; employment status; self-rated health status; cancer, chronic lung diagnosis, cardiac disease, or cerebrovascular disease diagnoses; and insurance status.

*: 0.05, **: 0.01, ***: <0.001
